# The clustering of physical health conditions and associations with co-occurring mental health problems and problematic alcohol use: a cross-sectional study

**DOI:** 10.1186/s12888-023-04577-3

**Published:** 2023-02-06

**Authors:** Katalin Ujhelyi Gomez, Orla McBride, Emmert Roberts, Colin Angus, Katherine Keyes, Colin Drummond, Iain Buchan, Kate Fleming, Ian Gilmore, Kim Donoghue, Laura Bonnet, Laura Goodwin

**Affiliations:** 1 Department of Primary Care and Mental Health, University of Liverpool, Waterhouse Block B 1St Floor, 1-5 Brownlow St, Liverpool, L69 3G UK; 2 School of Psychology, Ulster University, Belfast, UK; 3 National Addiction Centre, Institute of Psychiatry, Psychology and Neuroscience, King’s College London and South London and the Maudsley NHS Foundation Trust, London, UK; 4 School of Health and Related Research, University of Sheffield, Sheffield, UK; 5 Department of Epidemiology, Columbia University, New York, USA; 6 Department of Public Health, Policy, and Systems, University of Liverpool, Liverpool, UK; 7 National Disease Registration Service, NHS Digital, Leeds, UK; 8 Liverpool Centre for Alcohol Research, University of Liverpool, Liverpool, UK; 9 Clinical, Education & Health Psychology, University College London, London, UK; 10 Department of Health Data Science, University of Liverpool, Liverpool, UK; 11 Division of Health Research, Lancaster University, Lancaster, UK

**Keywords:** Multimorbidity, Mental health, Alcohol, Physical health, Latent class analysis

## Abstract

**Background:**

There is strong evidence for the co-occurrence of mental health conditions and alcohol problems, yet physical health outcomes among this group are not well characterised. This study aimed to identify clusters of physical health conditions and their associations with mental health and problematic alcohol use in England’s general population.

**Methods:**

Cross-sectional analysis of the 2014 Adult Psychiatric Morbidity Survey (*N* = 7546) was conducted. The survey used standardised measures of problematic alcohol use and mental health conditions, including the Alcohol Use Disorders Identification Test (AUDIT) and the Clinical Interview Schedule-Revised. Participants self-reported any lifetime physical health conditions. Latent class analysis considered 12 common physical illnesses to identify clusters of multimorbidity. Multinomial logistic regression (adjusting for age, gender, ethnicity, education, and occupational grade) was used to explore associations between mental health, hazardous drinking (AUDIT 8 +), and co-occurring physical illnesses.

**Results:**

Five clusters were identified with statistically distinct and clinically meaningful disease patterns: ‘*Physically Healthy’* (76.62%), *‘Emerging Multimorbidity’* (3.12%), ‘*Hypertension & Arthritis’* (14.28%), *‘Digestive & Bowel Problems’’* (3.17%), and ‘*Complex Multimorbidity’* (2.8%). Having a mental health problem was associated with increased odds of *‘Digestive & Bowel Problems’* (adjusted multinomial odds ratio (AMOR) = 1.58; 95% CI [1.15–2.17]) and *‘Complex Multimorbidity’* (AMOR = 2.02; 95% CI [1.49–2.74]). Individuals with co-occurring mental health conditions and problematic alcohol use also had higher odds of *‘Digestive & Bowel Problems’* (AMOR = 2.64; 95% CI [1.68–4.15]) and *‘Complex Multimorbidity’* (AMOR = 2.62; 95% CI [1.61–4.23]).

**Conclusions:**

Individuals with a mental health condition concurrent with problematic alcohol use experience a greater burden of physical illnesses, highlighting the need for timely treatment which is likely to include better integration of alcohol and mental health services.

**Supplementary Information:**

The online version contains supplementary material available at 10.1186/s12888-023-04577-3.

## Background

Multimorbidity is commonly defined as the co-existence of two or more long-term conditions [1] and can include mental and physical health conditions and problematic alcohol use [2]. Individuals who experience multimorbidity are characterised by lower levels of well-being and quality of life [3, 4], greater use of healthcare services [5, 6], and contribute disproportionately to healthcare costs [7, 8].

Physical and mental health conditions commonly co-occur [9–14] One in six (17%) people in England meet the criteria for a common mental disorder (CMD), e.g., depression and anxiety in the previous week [15], with differences by characteristics and demographic region [16]. Those with mental health conditions report poorer physical health compared to those without [15]. Specifically, there is a 10-year reduced life expectancy of those with a CMD or severe mental illness (SMI) [17]. Previous examination of the link between mental and physical conditions using the 2014 Adult Psychiatric Morbidity Survey (APMS) found more severe CMD among those with concurrent, long-term physical illness [18].

Nearly 20% of adults drink at a level that is potentially hazardous to health and many mental and physical health conditions are partially or wholly attributable to alcohol [15, 19–22]. One in five patients in UK hospitals use alcohol in a way that harms mental and/or physical health and one in 10 are dependent on alcohol [19]. Problematic alcohol use often co-occurs with other mental health conditions [23] with 86% of people using alcohol treatment services reporting a co-occurring mental health condition [24]. Alcohol affects multiple body systems causing harm such as liver problems, cardiovascular diseases, and cancers [25]. There is an increased prevalence of physical health conditions in those with a mental health problem explained by poorer health behaviours (e.g. alcohol use) combined with side effects of the long-term use of psychotropic medications and the impact of stress on the hypothalamic–pituitary–adrenal axis and inflammatory systems [26]. Therefore, we hypothesise that physical multimorbidity will be more common in those with co-occurring mental health conditions and problematic alcohol use compared to those with only one condition.

Structural determinants influence inequalities which may lead to poorer mental health [27] and health risking behaviours [28]. Research has shown that the most common disease cascades in individuals of lower socioeconomic status (SES) start with mental health and/or substance use problems [29]. A recent study also found that a multimorbidity cluster including mental health conditions and problematic alcohol use was associated with the greatest risk of premature mortality [30]. There is currently little research examining multimorbidity in individuals with co-occurring mental health and alcohol use disorders, compared to those with neither condition, or to those with a mental health condition alone. Existing research focusing on multimorbidity has typically examined which factors are associated with the number of health problems rather than defining clusters of disease. Such research has identified the important role of mental health in predicting multimorbidity [31], however, it may be helpful for clinicians to understand the types of physical health conditions that are most strongly associated with co-occurring disorders.

To address this gap, we used the 2014 APMS, a national survey conducted in England, to investigate the most common patterns of physical multimorbidity, and the associations with mental health problems and hazardous drinking. This allowed us to determine from cross-sectional analyses whether physical health is poorer in individuals with co-occurring mental health problems and hazardous drinking, compared to those with neither problem. This study aims 1) to explore the prevalence of self-reported physical non-communicable diseases (NCDs), comparing individuals who met criteria for a) a mental health problem (MHP) alone, b) hazardous drinking (HD) alone (Alcohol Use Disorder Identification Test (AUDIT) 8 + including hazardous, harmful alcohol use, and probable dependence), c) co-occurring MHP and HD, or d) neither, 2) to identify the most common clusters of physical NCDs and 3) to examine how the prevalence of these clusters compares across the aforementioned four mental health and alcohol use categories.

## Methods

### Study design

Secondary data analysis was performed on the 2014 APMS, the methods of which have been previously described elsewhere [32]. The APMS is a cross sectional survey of the prevalence of the general population’s mental health and treatment access in England in adults aged 16 + [15]. It employs a stratified, multistage random probability sampling design providing a representative sample of people living in private households [33]. The survey has been conducted every seven years since 1993. This study involved secondary data analysis of its fourth wave. The data was accessed with special permission from NHS Digital (Request number: DARS-NIC-220105-B3Z3S-v1.5). The study protocol and analysis plan were pre-registered on the Open Science Framework (https://osf.io/ewm9d/).

### Measures

#### Assessment of alcohol use

Past year alcohol consumption was measured by the AUDIT [34] including two prior screening questions: *“Do you ever drink alcohol nowadays?”*, a “No” response triggered an additional question “*Could I just check, does that mean you never have an alcoholic drink nowadays, or do you have an alcoholic drink very occasionally, perhaps for medicinal purposes or on special occasions like Christmas or New Year?*”. Participants who responded “No” did not complete the AUDIT. An AUDIT score of 0 or a negative response to alcohol use screening questions indicated ‘non-drinkers’. An AUDIT score of 1–7 indicated “low risk”, while a score of 8 + was considered as hazardous drinking (“hazardous/harmful use or probable dependence”). A binary ‘hazardous drinking’ variable was created including two categories: AUDIT 0–7 = non/low risk drinking, AUDIT ≥ 8 = hazardous drinking.

#### Assessment of mental health

CMDs included depression, anxiety, and social phobia and were measured by the Clinical Interview Schedule-Revised. Cases for mild, moderate and severe depression were grouped into an overall “depression” category to overcome the small cell sizes. Generalised anxiety disorder, obsessive compulsive disorder and panic disorder cases were clustered as “anxiety”, and social phobia, specific phobia and agoraphobia were classed as “phobia”. Post-traumatic stress disorder (PTSD) was assessed by the PTSD Checklist (PCL-C) corresponding to symptoms of DSM-IV PTSD in the past month [35].

Bipolar disorder and psychotic disorders are often grouped in the category of SMIs [36]. Bipolar disorder was screened by the 13-item Mood Disorder Questionnaire assessing manic/hypermanic symptoms and their co-occurrence over the lifetime causing moderate to severe problems (Cronbach’s α = 0.97) [37]. The five-item Psychosis Screening Questionnaire (PSQ) screened for symptoms of probable psychosis [38].

Other psychological problems included borderline personality disorder (BPD) and antisocial personality disorder (ASPD) assessed via the Structured Clinical Interview for DSM-IV Personality Disorders (SCID-II) over the lifetime [39]. Finally, attention-deficit hyperactivity disorder (ADHD) was measured by the six-item Adult Self-Report Scale [40].

#### Assessment of physical health

Participants self-reported (yes/no) the occurrence of 21 health conditions (e.g. cancer, diabetes, stroke) since the age of 16 years [18]. This measure allowed the exploration of links between mental health, alcohol use and lifetime physical health conditions rather than only considering current physical conditions that occurred in the previous 12 months. In this study, 12 NCDs were considered: cancer, diabetes, epilepsy/seizures, stroke, heart attack/angina, high blood pressure (hypertension), bronchitis/emphysema, asthma, stomach ulcer/digestive problems, liver problems, bowel/colon problems, and arthritis. Selection of these health conditions was based on previous frameworks of multimorbidity [41, 42] and the categorisation was further amended following focus groups with general practitioners (GPs) who suggested that some conditions should be dropped. Therefore, ‘migraine or frequent headaches’, ‘cataracts/eyesight problems’, ‘bone/back/joint or muscle problems’, and ‘skin problems’ were not included in the analyses due to the lack of information regarding their chronicity.

#### Demographic and socioeconomic characteristics

Potential confounding variables included gender, age, ethnicity, and assessment of socioeconomic position through education, occupational grade*,* and housing tenancy [15]. Supplemental Table 1 (ST1) includes further details on the variables used.

### Data analysis

Levels of missing data were low for most variables (range 0.01%—5.5%), with the highest proportion evident for the AUDIT (3.7%), PTSD (5.3%), and bipolar disorder (5.5%) measures. Complete case analysis was conducted, reducing the sample according to the completeness of the variables included in specific analyses.

#### Associations of physical NCDs with the mental health and alcohol use categories (aim 1)

Logistic regression analysis was conducted using Stata 14.0 to examine the associations of the mental health and alcohol use categories with the physical NCDs, (*N* = 7110) reporting odds ratios (OR) and 95% Confidence Intervals (CI). Sampling weights were applied to account for selection probabilities and non-response. The outcome variables were presence of any physical NCDs, while the predictor was mental health and alcohol use status: a) Any MHP only, b) HD only (AUDIT 8 +), c) co-occurring MHPs and HD, or d) neither (with all individuals assigned to a single category). Results were adjusted for age, gender, ethnicity, education, and occupational grade.

#### Physical NCDs in the general population (aim 2)

Binary data were analysed by exploratory Latent Class Analysis (LCA) conducted in MPlus 8.5 to identify clusters of physical NCDs. Maximum conditional probability estimated the number of underlying homogeneous classes (clusters) of 12 physical NCDs. Decisions in relation to the best fitting latent class model was guided by statistical fit indices and conceptual and clinical considerations. The Akaike Information Criterion (AIC) [43], the Bayesian Information Criterion (BIC) [44], the sample-size-adjusted BIC (SSABIC) [45], the Lo–Mendel–Rubin Likelihood Ratio Test (LMR-LRT) [46] and entropy were used [47]. Lower values on the AIC, BIC and the SSABIC reflect a good-fitting latent class model. A non-significant LRT value (*p* > 0.05) suggests that the model with one less class is a better explanation of the data. The entropy statistics ranging between 0 and 1 measures the accuracy of classification of individuals into clusters according to their model-based posterior probabilities with higher values reflecting better classification of participants. The maximum likelihood estimation method was employed when analysing ordinal observed variables.

#### Associations of the multimorbidity classes with the mental health and alcohol use categories (aim 3)

Multinomial logistic regressions (MLR) in MPlus 8.5 were conducted with a designated reference class to determine how the mental health and alcohol use categories were associated with class membership. Results report unadjusted multinomial OR (MOR) and adjusted MORs (AMOR) with 95% CIs controlling for age, gender, ethnicity, education, and occupational grade.

#### Sensitivity analyses

Previous research has demonstrated that individuals with mental health conditions have a higher risk of developing multimorbidity [18]. Therefore, the MLR analysis was repeated using the MHPs alone group as the reference group to explore whether the group with co-occurring MHPs and HD had a further increased risk. Additionally, MLR in MPlus investigated the association between type of mental health problem (i.e. CMD or SMI) with class membership, with ‘No CMD’ and ‘No SMI’ as reference categories.

## Results

### Characteristics of the overall sample

The full sample included 7546 individuals with 436 missing data primarily due to missingness on the AUDIT variable (*n* = 328) with some missing cases on the mental health variables (*n* = 108). Most of the full sample were female (51.07%), white ethnicity (87.7%), between the ages of 16 and 55 (65%), married or living in a partnership (49.34%), not working or have never worked (35.8%), in managerial/professional position (26.6%), had General Certificate of Secondary Education (GCSE)/A-Level (44.06%) or degree or higher-level education (33.75%), and owned their house (64.3%). In terms of the four groups defined by MHPs and HD, 61.69% (*N* = 4517) of the sample had neither a MHP nor were drinking at a hazardous level, 18.32% (*N* = 1341) had a MHP without HD, 13.04% (*N* = 817) drank at a hazardous level but had no MHPs, and 6.95% (*N* = 435) accounted for those who had both a MHP and HD. Details are included in Table 1.

**Table 1:** Participant characteristics by mental health problem and hazardous drinking status (*N* = 7110)

	Total N(%)	No MHP/No HD	**MHP**/No HD	No MHP/**HD**	**MHP/HD**
	*N* = 7110	N (weighted %, 95% CI) *N* = 4517	N (weighted %, 95% CI) *N* = 1341	N (weighted %, 95% CI) *N* = 817	N (weighted %, 95% CI) *N* = 435
**Demographic characteristics**					
*Gender*					
Male	2887 (48.93)	1750 (58.59, 56.36—60.78)	412 (14.78, 13.31—16.37)	496 (17.83, 16.20–19.59)	229 (8.81, 7.63–10.14)
Female	4223 (51.07)	2767 (64.66, 62.87–66.41)	929 (21.71, 20.35–23.14)	321 (8.44, 7.53–9.45)	206 (5.18, 4.42–6.07)
*Age*					
16–34 years	1544 (31.60)	836 (53.03, 50.10–55.94)	347 (20.82, 18.80–22.99)	201 (15.09, 13.06–17.38)	160 (11.06, 9.25–13.18)
35–54 years	2382 (33.96)	1397 (60.06, 57.76–62.31)	518 (19.77, 17.96–21.71)	295 (13.27, 11.76–14.93)	172 (6.91, 5.86–8.13)
55–74 years	2291 (25.26)	1528 (66.70, 64.38–68.93)	381 (15.98, 14.31–17.80)	288 (13.33, 11–76-15.09)	93 (3.99, 3.23–4.93)
75 + years	893 (8.88)	755 (8.43, 81.24–87.0)2	95 (10.63, 8.38–13.39)	33 (3.98, 2.83–5.58)	10 (1.05, 0.59–1.87)
*Marital status*					
Single	2050 (34.47)	1054 (50.52, 47.93–53.11)	484 (21.65, 19.86–23.54)	295 (16.50, 14.49–18.73)	217 (11.33, 9.67–13.25)
Married/Partnership	3221 (49.34)	2247 (68.34, 66.53–70.10)	494 (15.46, 14.11–16.91)	349 (11.62, 10.45–12.90)	131 (4.58, 3.82–5.49)
Separated/Divorced/Widowed	1838 (16.19)	1215 (65.17, 62.38–67-86)	363 (19.97, 17.92–22.19)	173 (9.99, 8.45–11.80)	87 (4.86, 3.93–5.99)
*Ethnicity*					
White	6442 (87.66)	4080 (60.90, 59.41–62.37)	1165 (17.44, 16.39–18.53)	791 (14.34, 13.32–15.43)	406 (7.32, 6.55–8.17)
Black / African / Caribbean	182 (3.06)	109 (64.40, 47.45–78.38)	52 (26.82, 14.69–43.8)3	8 (2.90, 0.76–10.50)	13 (5.87, 2.14–15.10)
Asian / Asian British	325 (6.80)	236 (72.00, 63.97–78.83)	74 (22.32, 16.82–29.01)	8 (2.80, 0.68–10.74)	7 (2.88, 0.49–15.27)
Mixed /Multiple ethnicity	138 (2.45)	82 (60.25, 41.58–76.34)	40 (26.51, 13.28–45.95)	9 (8.06, 3.27–18.53)	^a^
**SES characteristics**					
*Education*					
Degree or above	2320 (33.75)	1499 (63.21, 60.61–65.73)	370 (15.37, 13.77–17.12)	318 (15.34, 13.66–17.19)	133 (6.08, 4.98–7.41)
GCSE/A-Level	2838 (44.06)	1700 (57.88, 55.59–60.14)	582 (20.08, 18.41–21.87)	347 (13.71, 12.16–15.42)	209 (8.32, 7.13–9.70)
Foreign qualification	244 (2.98)	182 (73.30, 66.30–79.30)	34 (12.86, 8.47–19.05)	22 (11.02, 7.87–15.21)	^a^
No qualification	1641 (19.22)	1096 (66.06, 63.22–68.80)	338 (20.01, 17.88–22.33)	124 (7.98, 6.53–9.71)	83 (5.95, 4.64–7.60)
*Occupational grade*					
Managerial/Professional	1752 (26.64)	1076 (60.74, 58.01–63.40)	263 (13.80, 12.15–15.62)	289 (17.83, 15.86–19.98)	124 (7.64, 6.29–9.26)
Intermediate/Small employers and own account workers	1061 (15.62)	669 (61.53, 57.59–65.32)	205 (18.51, 15.60–21.81)	115 (11.94, 9.58–14.79)	72 (8.02, 6.11–10.47)
Lower supervisory and technical/semi-routine/routine	1288 (21.95)	763 (57.28, 53.67–60.82)	234 (17.47, 15.14–20.08)	194 (17.23, 14.51–20.34)	97 (8.02, 6.11–10.44)
Never worked/not worked in last year/not classified for other reason	2969 (35.79)	1989 (65.30, 63.17–67.38)	626 (22.02, 20.33–23.80)	215 (7.42, 6.38–8.62)	139 (5.26, 4.29–6.42)
*Housing tenancy*					
Owner-occupier	4661 (64.27)	3203 (66.26, 64.63–67.85)	678 (14.41, 13.35–15.54)	565 (13.76, 12.62–14.99)	215 (5.57, 4.80–4.46)
Social renter	1153 (15.42)	605 (52.92, 48.95–56-85)	371 (31.63, 28.05–35.44)	80 (7.15, 5.38–9.44)	97 (8.31, 6.37–10.76)
Private/other renter	1252 (20.30)	684 (53.99, 50.25–57.68)	277 (20.25, 17.76–22.98)	170 (15.49, 12.96–18.40)	121 (10.28, 8.25–12.74)

*SES* Socioeconomic status, *MHP* Mental Health Problem, *HD* Hazardous Drinking (AUDIT Score ≥ 8); ^a^*n* < 8: in line with NHS Digital reporting guidelines, numbers and percentages are not reported. Weights accounted for selection probabilities and non-response. There were 328 missing cases in the AUDIT data and 108 missing cases in the mental health data

### Demographics and SES by mental health and hazardous drinking status

Females, younger people (16–34 years), those living alone (single or separated/divorced/widowed), those from a non-white background, with an GCSE/A-Level qualification or no qualification, those not working/never worked, and social renters were more likely to have a MHP alone. HD alone was more likely among men, younger people (16–34), single, white, living in privately rented accommodation, with a degree or above, and working in managerial or lower supervisory positions. Co-occurring MHPs and HD were more likely among men, younger people (16–34), those single, white, living in privately rented accommodation, with an A level/GCSE education and working in intermediate or lower supervisory positions. For more details, refer to Table 1.

### Associations of physical NCDs with the mental health and alcohol use categories (aim 1)

After adjustment, all NCDs, apart from cancer, were significantly associated with having MHPs, with approximately 1.5-fold increased odds for diabetes, hypertension, and asthma, and 2–3-fold increased odds for heart attack/angina, stomach ulcer/digestive problems, arthritis, epilepsy/fits, stroke, bronchitis/emphysema, bowel/colon problems and liver problems (see ST 2). Following adjustment, HD alone was significantly associated with hypertension with nearly 1.5-fold increased odds. Additionally, individuals with co-occurring MHPs and HD had approximately 2-fold increased odds of hypertension, bronchitis/emphysema, stomach ulcer/digestive problems, heart attack/angina and bowel/colon problems, and a 6-fold increase in the odds of liver problems compared with individuals with neither MHP or HD (see Table ST2).

### Physical NCDs in the general population (aim 2)

#### Latent class estimation

To identify latent classes, seven latent class models were estimated (*N* = 7543). The goodness-of-fit statistics to identify the best fitting model are shown in Table ST3. There was a decrease in AIC throughout the six models. The BIC and SSABIC decreased to the four-class model and increased for the further models. The highest entropy statistics was found in the four-class model. Based on the goodness-of-fit indices, data could be well explained by a latent class model with three to six classes. The non-significant LMR-LRT statistics (*P* > 0.05) in the five-class model suggested that this model is not favourable to the four-class model. Additionally, while entropy with values closer to 1 (> 0.80) demonstrate clear delineation of classes [48], poor entropy is more difficult to specify as the quality of classification has different impact in different settings and even poor entropy can clearly differentiate some of the classes [49]. Although the entropy was slightly lower in the five-class model compared to the four-class model and the non-significant LRT value (*p* > 0.05) suggests that the four-class model is statistically a better explanation of the data, we chose the five-class model as it provided a clinically more informative and interpretable explanation of the clustering of physical health conditions following discussion with GPs.

#### Characteristics of the five-class model

**Fig. 1:**
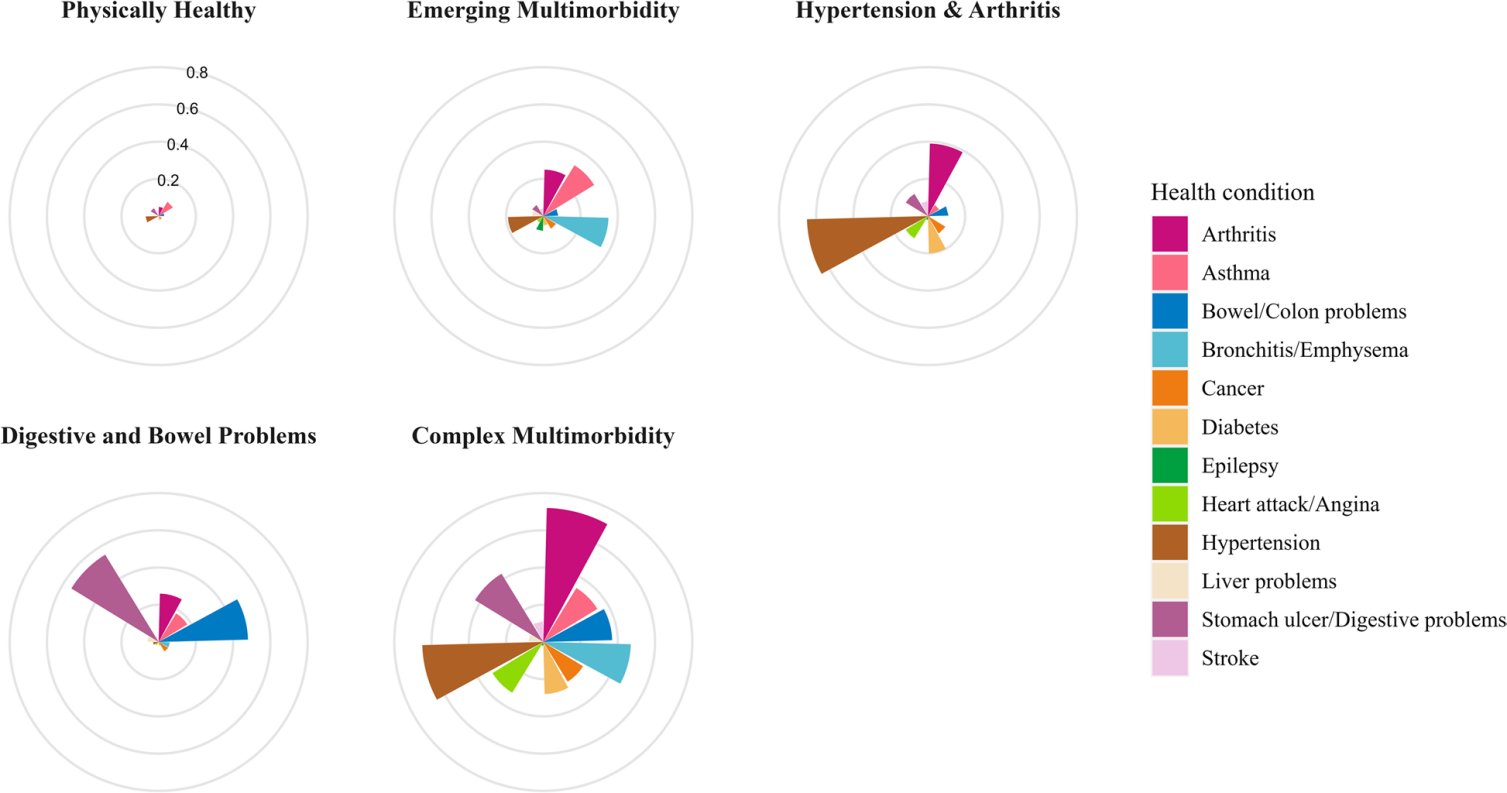
Profile plot for the 5-class model

Conditional probabilities are presented in Table 2 and a profile plot for the 5-class model is shown in Fig. 1. The largest class accounted for 76.6% of participants. It was labelled the ‘*Physically Healthy*’ class as the probabilities for reporting 10 out of 12 physical health conditions were ≤ 5%. There was a 7% likelihood of hypertension and 9% likelihood of asthma. The second largest class, called ‘*Hypertension & Arthritis’*, accounted for 14.3% of participants. In this class, there was a 65% probability for hypertension and an increasing probability of having arthritis (39%) and diabetes (20%). The third class named ‘*Digestive & Bowel Problems’* comprised 3.2% of participants, in which the probability of reporting stomach ulcer/digestive problems was 55% and bowel problems was 48%, with an increasing probability of arthritis (26%). The fourth class named ‘*Emerging Multimorbidity’* included 3.1% of individuals with an increasing probability of bronchitis/emphysema (35%), asthma (32%), and arthritis (25%). The fifth and final class accounted for 2.8% of individuals with ‘*Complex Multimorbidity’* with a probability of 72% for having arthritis, 65% for having hypertension, 47% for bronchitis/emphysema, and 43% for stomach ulcer/digestive problems. This class was also characterised by an increasing probability of other conditions: bowel problems (37%), asthma (34%), diabetes (28%), and cancer (25%).

**Table 2:** Class probabilities including number of cases and weighted percentages for each class (*N* = 7543)

Health condition	Physically Healthy	Emerging Multimorbidity	Hypertension & Arthritis	Digestive & Bowel Problems	Complex Multimorbidity
	*N* = 5780 (76.62%)	*N* = 235 (3.12%)	*N* = 1077 (14.28%)	*N* = 239 (3.17%)	*N* = 211 (2.8%)
**Cancer**					
Not present	0.98	0.92	0.89	0.94	0.75
Present	0.02	0.08	0.11	0.06	*0.25*
**Diabetes**					
Not present	0.98	0.95	0.80	0.98	0.72
Present	0.02	0.05	*0.20*	0.02	*0.28*
**Epilepsy**					
Not present	1.00	0.92	0.98	1.00	0.98
Present	0.00	0.08	0.02	0.00	0.02
**Stroke**					
Not present	1.00	0.96	0.92	1.00	0.89
Present	0.00	0.04	0.08	0.00	0.11
**Heart attack/angina**					
Not present	1.00	0.96	0.86	0.98	0.68
Present	0.00	0.04	0.14	0.02	*0.32*
**Hypertension**					
Not present	0.93	0.81	0.35	0.97	0.35
Present	0.07	0.19	**0.65**	0.03	**0.65**
**Bronchitis/emphysema**					
Not present	1.00	0.65	0.99	0.94	0.53
Present	0.00	*0.35*	0.01	0.06	**0.47**
**Asthma**					
Not present	0.91	0.68	0.93	0.82	0.66
Present	0.09	*0.32*	0.07	0.18	*0.34*
**Stomach ulcer/digestive problems**					
Not present	0.95	0.93	0.86	0.45	0.57
Present	0.05	0.07	0.14	**0.55**	**0.43**
**Liver problems**					
Not present	1.00	0.94	0.96	0.94	0.92
Present	0.00	0.06	0.04	0.06	0.08
**Bowel/colon problems**					
Not present	0.97	0.92	0.89	0.52	0.63
Present	0.03	0.08	0.11	**0.48**	*0.37*
**Arthritis**					
Not present	0.95	0.75	0.61	0.74	0.28
Present	0.05	*0.25*	*0.39*	*0.26*	**0.72**

Largest probabilities are bolded and *increasing probabilities* are italicised. There were three missing cases in physical health conditions

### Demographic and socioeconomic characteristics in the latent classes

There were more women in the ‘*Complex Multimorbidity’*, the ‘*Digestive & Bowel Problems’*, and the ‘*Emerging Multimorbidity’* classes, and more men in the ‘*Hypertension & Arthritis’* and ‘*Physically Healthy’* classes. Older people (55 +) were more likely to be classified in the ‘*Complex Multimorbidity*’, ‘*Digestive & Bowel Problems’*, and *‘Hypertension & Arthritis’* classes, while younger people were more likely to be classified as *‘Physically Healthy’*. The classes with higher probabilities of NCDs were more likely to include people who have been ‘separated/divorced/widowed’ compared to those ‘married/living in partnership’ or who were ‘single’. Similarly, white individuals were more likely to be in these classes with NCDs. People in the classes with NCDs were more likely to have no qualifications, although there were more people with degree or above attainment in the ‘*Digestive & Bowel Problems’* class. The ‘*Physically Healthy’* class included more individuals with GCSE or higher qualification. Individuals who were out of work and social renters were more likely to belong to the classes with physical NCDs, although the number of private/other renters was higher in the ‘*Digestive & Bowel Problems’* class. Table 3 includes details of these characteristics.

**Table 3:** Participant demographic characteristics by latent class (*N* = 7543)

	**Total N (weighted %)**	***‘Physically Healthy’ class*** **(*n* = 5364)**	***‘Emerging Multimorbidity’ class*** **(*n* = 273)**	***‘Hypertension & Arthritis’ class*** **(*n* = 1358)**	***‘Digestive & Bowel Problems’ class*** **(*n* = 274)**	***‘Complex Multimorbidity’ class*** **(*n* = 274)**
		N (weighted %) 95% CI	N (weighted %) 95% CI	N (weighted %) 95% CI	N (weighted %) 95% CI	N (weighted %) 95% CI
*Gender*						
Male	3058 (48.84)	2161 (74.54, 72.71–76.28)	115 (3.18, 2.58–3.92)	597 (16.91, 15.48–18.43)	93 (2.76, 2.20–3.45)	91 (2.62, 2.08–3.31)
Female	4488 (51.16)	3203 (73.63, 72.18–75.03)	158 (3.37, 2.88–3.95)	761 (15.17, 14.03–16.38)	181 (3.91, 3.37–4.53)	183 (3.93, 3.36–4.58)
*Age*						
16–34 years	1595 (30.99)	1311 (83.41, 81.28–85.36)	47 (2.83, 2.04–3.92)	145 (8.58, 7.18–10.23)	48 (2.84, 2.04–3.94)	44 (2.33, 1.71–3.16)
35–54 years	2474 (33.48)	1916 (78.02, 76.13–79.80)	89 (3.41, 2.71–4.28)	320 (13.20, 11.68–14.90)	87 (3.13, 2.52–3.89)	60 (2.24, 1.70–2.94)
55–74 years	2415 (25.58)	1559 (65.17, 63.04–67.25)	94 (3.38, 2.71–4.20)	553 (22.73, 20.86–24.72)	98(4.04, 3.26–4.99)	110 (4.68, 3.84–5.70)
75 + years	1062 (9.94)	578 (54.54, 51.08–57.96)	43 (3.98, 2.63–5.97)	340 (31.36, 28.02–34.91)	41 (3.87, 2.79–5.35)	60 (6.25, 4.63–8.38)
*Marital status*						
Single	2144 (33.96)	1655 (80.89, 78.91–82.73)	76 (3.08, 2.32–4.09)	269 (10.26, 8.97–11.70)	76 (3.13, 2.41–4.06)	66 (2.64, 2.01–3.46)
Married/Partnership	3383 (49.23)	2437 (72.72, 71.06–74.33)	108 (3.13, 2.56–3.83)	609 (17.56, 16.25–18.95)	117 (3.34, 2.74–4.05)	112 (3.25, 2.66–3.96)
Separated/Divorced/Widowed	2018 (16.81)	1271 (64.21, 61.83–66.53)	89 (4.10, 3.27–5.14)	480 (23.14, 21.13–25.29)	81 (3.81, 3.02–4.80)	96 (4.73, 3.81–5.85)
*Ethnicity*						
White	6813 (87.33)	4805 (73.29, 72.10–74.44)	249 (3.33, 2.90–3.82)	1255 (16.52, 15.55–17.54)	254 (3.54, 3.11–4.02)	247 (3.33, 2.90–3.81)
Non-white	705 (12.67)	535 (79.27, 75.00–82.98)	23 (2.88, 1.85–4.46)	101 (12.76, 9.57–16.82)	19 (1.96, 0.92–4.12)	27 (3.13, 1.71–5.64)
**SES characteristics**						
*Education*						
Degree or above	2410 (33.30)	1808 (77.07, 75.09–78.93)	79 (2.95, 2.27–3.83)	356 (13.51, 12.15–15.00)	90 (3.72, 2.92–4.73)	77 (2.75, 2.16–3.51)
A-level/GCSE	2939 (43.23)	2184 (76.78, 74.93–78.53)	112 (3.51, 2.86–4.32)	451 (13.87, 12.48–15.39)	99 (2.97, 2.39–3.69)	91 (2.87, 2.29–3.59)
Foreign qualification	272 (3.14)	181 (68.95, 59.03–77.40)	^a^	60 (20.50, 14.76–24.75)	8 (2.62, 0.60–10.74)	16 (5.90, 2.11–15.42)
No qualification	1843 (20.32)	1132 (64.29, 61.82–66.69)	73 (3.55, 2.73–4.60)	473 (23.73, 21.70–25.89)	74 (3.60, 2.75–4.69)	90 (4.83, 3.89–5.99)
*Occupational grade*						
Managerial/Professional	1795 (25.97)	1424 (80.22, 78.09–82.19)	60 (3.05, 2.25–4.11)	214 (11.55, 10.00–13.31)	59 (2.23, 2.41–4.30)	38 (1.96, 1.34–2.85)
Intermediate/Small employers and own account workers	1104 (15.45)	855 (78.55, 75.40–81.39)	36 (3.02, 1.98–4.59)	147 (13.21, 10.95–15.84)	29 2.39, 1.58–3.61)	36 (2.84, 1.83–4.39)
Lower supervisory and technical/semi-routine/routine	1342 (21.59)	1049 (79.60, 76.83–82.12)	49 (3.50, 2.47–4.93)	167 (11.59, 9.67–13.82)	49 (3.29, 2.33–4.62)	27 (2.03, 1.25–3.28)
Never worked/not worked in last year/not classified for other reason	3258 (37.00)	1999 (64.62, 62.70–66.50)	127 (3.43. 2.82–4.17)	824 (22.94, 21.38–24.58)	135 (3.85, 3.18–4.66)	172 (5.15, 4.39–6.05)
*Housing tenancy*						
Owner-occupier	4921 (63.98)	3489 (73.62, 72.19–75.01)	178 (3.30, 2.82–3.86)	911 (16.61, 15.48–17.79)	172 (3.20, 2.71–3.76)	170 (3.27, 2.76–3.88)
Social renter	1270 (15.97)	859 (71.31, 68.04–74.37)	48 (3.74, 2.53–5.49)	251 (17.39, 14.90–20.19)	48 (3.28,2.33–4.61)	63 (4.29, 3.22–5.69)
Private/other renter	1304 (20.05)	978 (77.74, 74.75–80.46)	45 (2.85, 1.89–4.26)	189 (13.11, 10.97–15.60)	53 (3.90, 2.82–5.36)	38 (2.41, 1.71–3.37)

*SES* Socioeconomic status. ^a^*n* < 8: in line with NHS Digital reporting guidelines, numbers and percentages are not reported. Non-white: Black, Asian, and mixed ethnicities were combined and labelled ‘non-white’ due to small cell sizes. There were three missing cases in physical health conditions

### Associations of the multimorbidity classes with the mental health and hazardous drinking categories (aim 3)

**Fig. 2:**
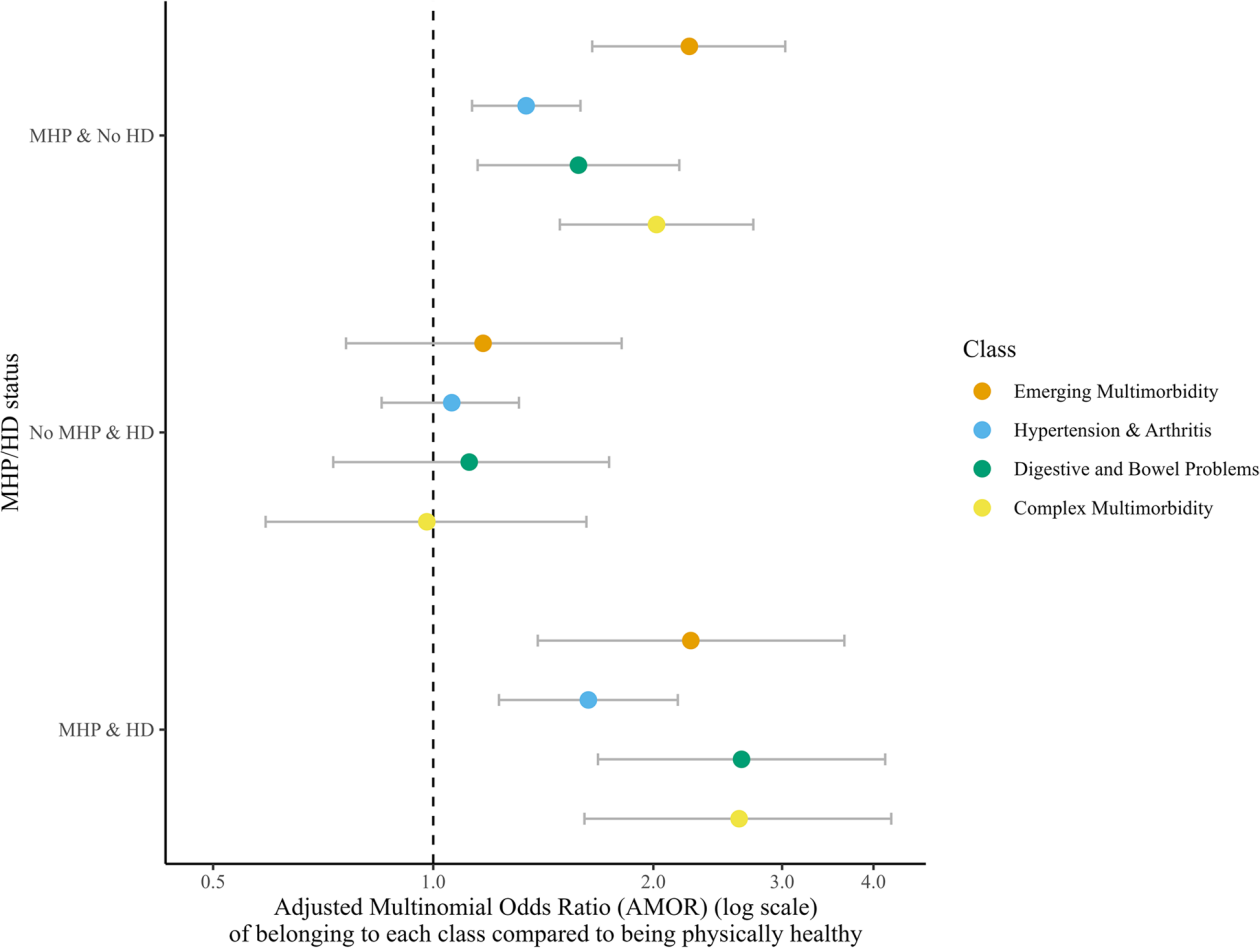
Associations of the multimorbidity classes with the mental health and hazardous drinking categories

As presented in Fig. 2., after adjustment, individuals with a MHP alone had around twice the odds of being classified in the *‘Emerging Multimorbidity’* and the *‘Complex Multimorbidity’* classes and 1.5-fold increased odds of being in the *‘Digestive & Bowel Problems’* class (rather than the *'Physically Healthy'* class), compared to the group with no MHPs and no HD. There were no statistically significant associations with the classes for the HD only group. Those with co-occurring MHPs and HD had over 1.5-fold increased odds of being classified in the *‘Hypertension & Arthritis’* class, over twice the odds of being assigned to the *‘Emerging Multimorbidity’* class and 2.5-fold increased odds of being categorised in the *‘Digestive and Bowel Problems’* and *‘Complex Multimorbidity’* classes (rather than the *'Physically Healthy'* class), compared to the group without MHPs and HD. ST 4 provides detailed information on the unadjusted and adjusted associations.

#### Sensitivity analysis

When conducting the MLR analysis with the MHPs alone group as the reference group, and after adjustment, those with co-occurring MHPs and HD had 1.7-fold increased odds of being assigned to *‘Digestive & Bowel Problems’* class compared to those with MHPs alone (Table 4).

**Table 4:** Multinomial logistic regression showing the associations between mental health problem/hazardous drinking status and the latent classes of multimorbidity (*N* = 7107). Reference group: MHP & No HD

	***‘Physically Healthy’ class*** ***(n* = *5078)***	***‘Emerging Multimorbidity’ class*** ***(n* = *256)***		***‘Hypertension & Arthritis’ class*** ***(n* = *1266)***		***‘Digestive and Bowel Problems’ class*** ***(n* = *257)***		***‘Complex Multimorbidity’ class*** ***(n* = *250)***	
**MHP/HD status**	**N (%)**	**N (%)** **MOR (95% CI)**	**AMOR (95% CI)**	**N (%)** **MOR (95% CI)**	**AMOR (95% CI)**	**N (%)** **MOR (95% CI)**	**AMOR (95% CI)**	**N (%)** **MOR (95% CI)**	**AMOR (95% CI)**
**NO MHP & NO HD** **(*n* = 4517)**	3282(72.66)	135 (2.99)		812 (17.98)		146 (3.23)		142 (3.14)	
		0.51***	0.45***	0.90	0.75***	0.69*	0.63**	0.56***	0.50***
		**(0.38–0.69)**	**(0.33–0.61)**	(0.77–1.06)	**(0.63–0.88)**	**(0.50–0.94)**	**(0.46–0.87)**	**(0.42–0.75)**	**(0.37–0.67)**
**MHP & NO HD** **(*n* = 1338)**	893 (66.74)	72 (5.38) 1.00		246 (18.39)		58 (4.33)		69 (5.16)	
			1.00	1.00	1.00	1.00	1.00	1.00	1.00
**NO MHP & HD** **(*n* = 817)**	616 (75.4)	27 (3.3)		129 (15.79)		27 (3.3)		18 (2.2)	
		0.51**	0.52**	0.76*	0.78	0.68	0.71	0.38***	0.48**
		**(0.35–0.86)**	**(0.33–0.84)**	**(0.60–0.96)**	(0.61–1.01)	(0.42–1.08)	(0.44–1.16)	**(0.22–0.64)**	**(0.28–0.83)**
**MHP & HD** **(*n* = 435)**	287 (65.98)	22 (5.06)		79 (18.16)		26 (5.98)		21 (4.83)	
		0.95	1.01	1.00	1.22	1.40	1.67*	0.95	1.30
		(0.58–1.56)	(0.61–1.68)	(0.75–1.33)	(0.90–1.65)	(0.86–2.26)	**(1.01–2.76)**	(0.57–1.57)	(0.77–2.18)

*MHP* Mental Health Problem, *HD* Hazardous Drinking (AUDIT Score ≥ 8); *p < .05; **p ≤ .01; ***p ≤ .005; *MOR* Multinomial Odds Ratio, *AMOR* Adjusted MOR; Percentages are weighted with APMS survey weights. There were 328 missing cases in the AUDIT data, 108 missing cases in the mental health data, and three missing cases in the NCD data

### Associations between the latent classes of multimorbidity and CMD/SMI

**Fig. 3:**
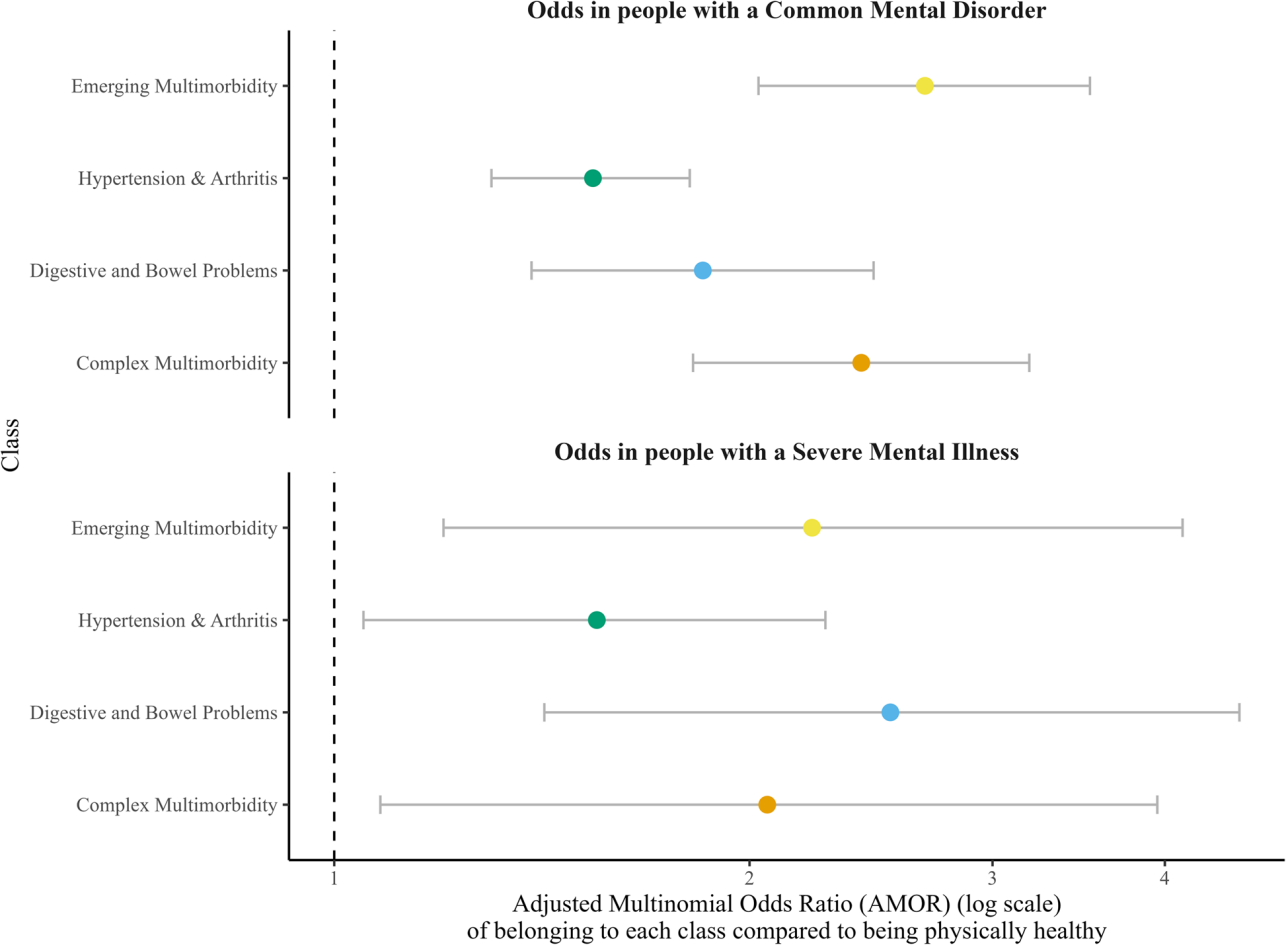
Associations between the latent classes of multimorbidity and CMD/SMI

As reflected in Fig. 3, following adjustment, individuals with a CMD had approximately 2.5-fold increased odds of being classified in the *‘Emerging Multimorbidity’* and *‘Complex Multimorbidity’* classes, nearly 2-fold increased odds of being classified in the *‘Digestive & Bowel Problems’* class, and over 1.5-fold increased odds of being assigned to the ‘*Hypertension & Arthritis’* class compared to those without a CMD. Associations were similar for SMI with 1.5-fold increased odds for being categorised in the *‘Hypertension & Arthritis’* class, approximately 2-fold increased odds of being classified in the *‘Emerging Multimorbidity’* and *‘Complex Multimorbidity’* classes, and around 2.5-fold increased odds to be assigned to the ‘*Digestive & Bowel Problems’ class*. The unadjusted and adjusted associations between the latent classes with the different mental health problems are reported in Table ST5.

## Discussion

This study aimed to investigate the most common patterns of physical multimorbidity, and their cross-sectional associations with MHPs and HD in the APMS 2014. Most of the physical NCDs were more common in individuals who had either MHPs alone or those with co-occurring MHPs and HD with a particularly strong association with liver problems for those with co-occurring problems. LCA identified five classes in this sample: *‘Physically Healthy’* (76.62%), *‘Hypertension & Arthritis’* (14.28%), *‘Digestive & Bowel Problems’* (3.17%), *‘Emerging Multimorbidity’* (3.12%), and *‘Complex Multimorbidity’* (2.8%). Individuals with MHPs only and those with co-occurring MHPs and HD were more likely to experience multimorbidity. It was only *‘Digestive and Bowel Problems’* for which the group with co-occurring problems had increased odds compared to those with MHPs only. Additionally, those drinking alcohol at hazardous levels (without co-occurring MHPs) did not have increased odds of multimorbidity, which is unexpected but may relate to the characteristics of this group.

This work supports findings of previous studies in relation to the association between MHPs and physical multimorbidity [11–14, 17, 18]. The NCDs included in the study (apart from cancer) were all significantly positively associated with having MHPs. However, despite extensive evidence for the association of problematic alcohol use and chronic physical health conditions [19–22], in the present study, HD alone was only associated with hypertension. Previous work also analysing data from the APMS 2014 found no association between problematic use of alcohol and the presence of chronic physical conditions (cancer, asthma, epilepsy, cardiovascular disease) [18]. This may be the result of the ‘sick quitter effect’ bias [50], as many people decide to abstain due to health concerns arising from alcohol or other conditions, and this effect has been evidenced across a range of age groups [51–53]. Additionally, a high proportion of the group with HD only were from a higher socioeconomic background, which is a protective factor against alcohol harms [54]. However, co-occurring MHPs and HD was associated with a number of NCDs, with the strongest association shown for liver problems. Several liver disorders are considered as wholly alcohol attributable conditions which may explain this increase in odds [55].

Compared to individuals with no MHPs and no HD, those with MHPs were more likely to experience multimorbidity, with increased odds of the following classes: ‘*Emerging Multimorbidity’*, ‘*Hypertension & Arthritis’*, ‘*Digestive & Bowel Problems’*, ‘*Complex Multimorbidity’*, demonstrating the link previously identified between mental and physical health [11–14, 17, 18]. Similarly to the results of the logistic regression, HD was not associated with physical multimorbidity, a contradiction to the well-known causal relationship between problematic alcohol use and physical health conditions [19, 21, 22]. However, there was a link between co-occurring MHPs and HD and the classes with the different levels of multimorbidity, with somewhat stronger association for the classes of ‘*Digestive & Bowel Problems’* and ‘*Complex Multimorbidity’*. Additionally, in comparison to the MHPs only group (sensitivity analysis), those with co-occurring MHPs and HD were more likely to be classified in the ‘*Digestive & Bowel Problems’* class with gastrointestinal disorders that are typical consequences of problematic alcohol use [56]. These findings indicate that alcohol use may increase the risk of alcohol attributable physical diseases in individuals with MHPs.

### Strengths and limitations

The APMS 2014 covers a wide range of physical health conditions and uses validated measures for both mental health problems and alcohol use, although many are self-reported, introducing some desirability bias [57]. The survey only includes individuals living in private households and excludes institutional settings, such as prisons, offender institutions or those who experience rough sleeping, where MHPs and substance use are more common, which may have contributed to the small number of these cases rendering it difficult to conduct sub-group analyses. Due to the cross-sectional survey design, it was not possible to examine causal relationships or the direction of the effect. Future research should investigate the associations found by this study longitudinally and determine if the ordering of the mental health and alcohol problems is important. Future work should examine transitions and timing of incident disorders to infer whether an individual’s physical multimorbidity risk changes were likely to have resulted from pre-existing mental health and alcohol use problems.

In terms of physical NCDs, the variable used referred to physical health conditions experienced since the age of 16 without restricting the analysis to health conditions that occurred only in the last 12 months. This enabled the exploration of physical health over the lifetime focusing on more chronic conditions but did not allow us to identify whether the illness reported had been diagnosed by a health professional, when the condition first occurred, whether it occurred in the previous 12 months, and whether the individual had received treatment or medication. This was a limitation of the question wording in the survey.

The lack of association between hazardous drinking and physical NCDs may be attributable to the way the MHPs and HD groups were created. Guided by the limited sample size, individuals were considered as hazardous drinkers if they scored ≥ 8 on the AUDIT including those who drink at an increasing risk level rather than only those with higher risk drinking or possible dependence. Higher risk drinking may have shown an association with other NCDs.

Finally, although survey weighting addressed selection probabilities and non-response to a certain extent to make the results representative of the target population, the sample had a predominantly white ethnicity, reducing the generalisability to other ethnic groups.

### Implications

People with co-occurring MHPs and HD are also more likely to experience physical multimorbidity, yet in practice services are not as integrated as they could be [58] highlighting the mismatch between healthcare needs and utilisation [59]. Addiction and mental health services are not currently equipped to screen for and identify chronic physical NCDs and therefore, refer individuals to other services but this can mean that people only get the support when symptoms and chronicity are more serious. As a result of multimorbidity and its poor clinical management, life expectancy is reduced and the personal, social and economic burden of illness across the lifespan is increased [60]. There are currently issues from a UK context with the separation and lack of communication within mental health, addiction, and acute physical health services [58]. This research, therefore, provides evidence on the need for integration of these services.

## Conclusion

Individuals with MHPs and co-occurring MHPs and HD are more likely to experience physical multimorbidity. Our findings highlight the need for mental health, alcohol and physical healthcare services to be better integrated to improve the management of multimorbidity. Furthermore, prevention of multimorbidity among people with co-occurring mental health and alcohol problems should be prioritised, particularly with a focus on gastrointestinal conditions.

## Supplementary Information

**Additional file 1: Supplemental Table 1 (ST1).** Measured variables. **Supplemental Table 2 (ST2).** Logistic regression showing the association between the physical health conditions and the mental health/hazardous drinking categories (*N*=7107). **Supplemental Table 3 (ST3).** Model fit criteria for latent class analysis including 12 physical health conditions (*N* = 7543). **Supplemental Table 4 (ST4).** Multinomial logistic regression showing the associations between mental health problem/hazardous drinking status and the latent classes of multimorbidity (*N*=7107). Reference group: No MHP & No HD. **Supplemental Table 5 (ST5).** Multinomial logistic regression showing the associations between CMD/SMI and the latent classes of multimorbidity (CMD *N*=7543; SMI *N*=7091 – SMI). **Supplemental Table 6 (ST6).** STROBE Checklist.

## Data Availability

Raw data were generated at NHS Digital and available through the Data Access Request Service (DARS) https://digital.nhs.uk/services/data-access-request-service-dars. Derived data supporting the findings of this study are available from the corresponding author [KUG] on request.
