# Supplementary material for: The clustering of physical health conditions and associations with co-occurring mental health problems and problematic alcohol use: a cross-sectional study
Source: BMC Psychiatry. 2023 Feb 6;23:89. doi: 10.1186/s12888-023-04577-3 (PMC9901006; doi:10.1186/s12888-023-04577-3)
Supplement: Supplementary file 1 — Additional file 1: Supplemental Table 1 (ST1). Measured variables. Supplemental Table 2 (ST2). Logistic regression showing the association between the physical health conditions and the mental health/hazardous drinking categories (N=7107). Supplemental Table 3 (ST3). Model fit criteria for latent class analysis including 12 physical health conditions (N = 7543). Supplemental Table 4 (ST4). Multinomial logistic regression showing the associations between mental health problem/hazardous drinking status and the latent classes of multimorbidity (N=7107). Reference group: No MHP & No HD. Supplemental Table 5 (ST5). Multinomial logistic regression showing the associations between CMD/SMI and the latent classes of multimorbidity (CMD N=7543; SMI N=7091 – SMI). Supplemental Table 6 (ST6). STROBE Checklist. [file 12888_2023_4577_MOESM1_ESM.docx]

**Online Supplementary Material**

Contents

**Tables**

Supplemental Table 1 (ST1): Measured variables

Supplemental Table 2 (ST2): Logistic regression showing the association between the physical health conditions and the mental health/hazardous drinking categories (N=7107)

Supplemental Table 3 (ST3): Model fit criteria for latent class analysis including 12 physical health conditions (N = 7543)

Supplemental Table 4 (ST4): Multinomial logistic regression showing the associations between mental health problem/hazardous drinking status and the latent classes of multimorbidity (N=7107). Reference group: No MHP & No HD.

Supplemental Table 5 (ST5): Multinomial logistic regression showing the associations between CMD/SMI and the latent classes of multimorbidity (CMD N=7543; SMI N=7091 – SMI).

Supplemental Table 6 (ST6): STROBE Checklist

**Supplemental Table 1:** Measured variables

| **Variable** | **Variable type** | **Measure** | **Specific classification(s)** |
| --- | --- | --- | --- |
| **Alcohol use** |  | Alcohol Use Disorder Identification Test (AUDIT)  Code DrinkAny | Non-drinker (AUDIT 0 or ‘No’ to screening questions)/Low risk (AUDIT 1-7)  Hazardous drinking (AUDIT 8+) (including *Hazardous use*/*Harmful use*/*probable dependence*) |
| **Common Mental Disorder (CMD)** | Depression | Revised Clinical Interview Schedule  (CIS-R) | All depression*  No depression |
|  | Anxiety | CIS-R | All anxiety*  No anxiety |
|  | Phobia | CIS-R | All phobia*  No phobia |
|  | CMD not otherwise specified (CMD_NOS) | CIS-R | CMD_NOS  No CMD_NOS |
|  | Post-traumatic Stress Disorder (PTSD) | PTSD Checklist (PCL-C) | PTSD**  No PTSD |
| **Serious Mental Illness (SMI)** | Bipolar disorder | Mood Disorder Questionnaire (MDQ) | Bipolar disorder  No bipolar disorder |
|  | Psychotic disorder | Psychosis Screening Questionnaire (PSQ) | Probable psychosis  No probable psychosis |
| **Other mental health problems** | Borderline personality disorder (BDPDS) | Structured Clinical Interview for DSM-IV Personality Disorders (SCID-II) | BDPDS  No BDPDS |
|  | Antisocial personality disorder (ASPDS) | SCID-II | ASPDS  No ASPDS |
|  | Attention-Deficit Hyperactivity Disorder (ADHD) | Adult Self-Report Scale | ADHD  No ADHD |
| **Physical health conditions in adulthood** | Health conditions grouped | Have you had any of these health conditions since the age of 16? | Yes/No |
| **Sociodemographic characteristics** | Gender |  | Male /Female |
|  | Age |  | 16-34 years old  35-54 years old  55-74 years old  75+ years old |
|  | Ethnicity |  | White/Non-white |
|  | Marital status |  | 1.Single  2.Married/same-sex civil partnership  3.Separated/Divorced/Widowed |
|  | Education | Highest education qualification | 1.Degree or above  2. A-level, GCSE or equivalent  3. Foreign qualification  4. No qualifications |
|  | Occupation | National Statistics Socio-economic Classification (NSSEC) | 1.Managerial/professional  2.Intermediate/small employers and own account  3.Lower supervisory and technical/semi-routine/routine  4.Never worked/not worked in last year/not classified for other reason |
|  | Housing | Tenure | 1.Owner-occupier  2.Social renter  3.Private/other renter |

*Cases for mild, moderate and severe depression were grouped into an overall “depression” category to overcome the small cell sizes. Generalised anxiety disorder, obsessive compulsive disorder and panic disorder cases were clustered as “anxiety”. Social phobia, specific phobia and agoraphobia were classed as “phobia”. **Meeting criteria for probable PTSD required a score of 50 on the 17-item PCL-C and positive responses to a minimum of one item on re-experiencing, three items on avoidance and numbing, and two on hyperarousal (Fear et al., 2016).

**Supplemental Table 2.** Logistic regression showing the association between the physical health conditions and the mental health/hazardous drinking categories (N=7107).

| **Physical health conditions** | **No MHP/**  **No HD**  **n=4515** | **MHP/No HD**  **n=1341** | | **No MHP/HD**  **n=816** | | **MHP/HD**  **n=435** | |
| --- | --- | --- | --- | --- | --- | --- | --- |
|  | **N(%) OR** | **N(%) OR**  **(95% CI)** | **AOR**  **(95% CI)** | **N(%) OR**  **95% CI** | **AOR**  **(95% CI)** | **N(%) OR**  **(95% CI)** | **AOR**  **(95% CI)** |
| Cancer | 328 (68.38)  1 | 91 (18.1)  0.89  (0.68-1.15) | 1.27  0.96-1.67 | 37 (8.02)  ***0.54******  ***(0.37-0.79)*** | 0.79  (0.53-1.17) | 27 (5.50)  0.70  (0.44-1.12) | 1.58  (0.97-2.57) |
| Diabetes | 338 (64.48)  1 | 120 (23.22)  1.23  (0.96-1.58) | **1.64*****  **1.24-2.18** | 35 (7.05)  ***0.50******  ***(0.33-0.76)*** | 0.70  (0.44 -1.11) | 20 (5.25)  0.71  (0.41-1.23) | 1.37  (0.76-2.44) |
| Epilepsy | 44 (47.37)  1 | 39 (35.33)  **2.55*****  **(1.50-4.33)** | **2.40*****  **1.39-4.15** | 10 (9.34)  0.93  (0.42-2.05) | 1.06  (0.48-2.38) | 8 (7.96)  1.50  (0.63-3.54) | 1.50  (0.61-3.68) |
| Stroke | 119 (57.27)  1 | 52 (28.93)  **1.73*****  **(1.22-2.44)** | **2.34*****  **1.60-3.42** | 16 (9.33)  0.77  (0.42-1.41) | 1.23  (0.62-2.40) | 9 (4.47)  0.69  (0.32-1.47) | 1.54  (0.67-3.53) |
| Heart attack/angina | 241 (62.75)  1 | 82 (22.79)  1.23  (0.92-1.65) | **1.96*****  **1.41-2.72** | 27 (7.48)  ***0.55****  ***(0.35-0.89)*** | 0.82  (0.49-1.37) | 21 (6.99)  0.99  (0.59-1.64) | **2.47***  **(1.43-4.26)** |
| Hypertension | 1108 (63.18)  1 | 326 (19.55)  1.05  (0.89-1.24) | **1.50*****  **1.24-1.80** | 187 (12.18)  0.89  (0.72-1.11) | **1.36***  **(1.06-1.74)** | 82 (5.09)  **0.67*****  **(0.51-0.87)** | **1.45****  **(1.09-1.93)** |
| Bronchitis/emphysema | 204 (51.11)  1 | 126 (31.09)  **2.14*****  **(1.65-2.77)** | **2.66*****  **2.03-3.51** | 37 (11.14)  1.03  (0.70-1.52) | 1.39  (0.91-2.13) | 29 (6.66)  1.16  (0.72-1.89) | **2.06****  **(1.23-3.43)** |
| Asthma | 501 (55.61)  1 | 243 (24.71)  **1.59*****  **(1.33-1.90)** | **1.57*****  **1.30-1.89** | 98 (12.34)  1.06  (0.82-1.37) | 1.13  (0.87-1.48) | 62 (7.34)  1.19  (0.85-1.68) | 1.20  (0.85-1.70) |
| Stomach ulcer/digestive problems | 464 (53.47)  1 | 250 (28.69)  **1.97*****  **(1.61-2.42)** | **2.24*****  **1.82-2.76** | 72 (9.26)  0.80  (0.60-1.08) | 0.93  (0.68-1.27) | 68 (8.58)  **1.49***  **(1.06-2.09)** | **2.05*****  **(1.45-2.90)** |
| Liver problems | 64 (38.79)  1 | 52 (31.11)  **2.76*****  **(1.80-4.22)** | **3.19*****  **2.06-4.93** | 12 (10.53)  1.29  (0.65-2.56) | 1.42  (0.71-2.83) | 27 (19.58)  **4.68*****  **(2.88-7.62)** | **6.14*****  **(3.71-10.15)** |
| Bowel/colon problems | 350 (51.65)  1 | 209 (30.70)  **2.16*****  **(1.75-2.67)** | **2.45*****  **1.96-3.07** | 50 (8.07)  0.73  (0.51-1.03) | 0.88  (0.61-1.27) | 56 (9.58)  **1.73*****  **(1.25-2.39)** | **2.47*****  **(1.76-3.47)** |
| Arthritis | 924 (64.45)  1 | 319 (23.19)  **1.26****  **(1.07-1.49)** | **1.87*****  **1.54-2.27** | 91 (6.96)  ***0.47******  ***(0.36-0.61)*** | *0.78*  *(0.58-1.04)* | 70 (5.41)  **0.71***  **(0.52-0.98)** | **1.98***  **(1.38-2.85)** |

MHP, Mental Health Problem; HD, Hazardous Drinking (AUDIT Score ≥8); *p<.05; **p≤.01; ***p≤.005; OR = unadjusted Odds Ratio; AOR = Adjusted Odds Ratio for age, gender, ethnicity, education, and occupational grade. There were 328 missing cases in the AUDIT data, 108 missing cases in the mental health data, and three missing cases in the NCD data.

**Supplemental Table 3.** Model fit criteria for latent class analysis including 12 physical health conditions (N = 7543).

| **Number of classes** | **Loglikelihood** | **Entropy** | **AIC** | **BIC** | **SSABIC** | **LMR-LRT**  **(p-value)** | **BVR (cut off 3.84)** | **Range of class probabilities** |
| --- | --- | --- | --- | --- | --- | --- | --- | --- |
| 1 | -22905.103 | N/A | 45834.205 | 45917.346 | 45879.212 | N/A | 63 | N/A |
| 2 | -21872.122 | 0.64 | 43794.245 | 43967.454 | 43888.009 | 2048.313 (0.00) | 5 | 0.85-0.92 |
| 3 | -21783.106 | 0.68 | 43642.211 | 43905.490 | 43784.734 | 176.513 (0.00) | 4 | 0.72-0.90 |
| 4 | -21723.291 | **0.70** | 43548.582 | **43901.930** | **43739.862** | **118.607 (0.00)** | 2 | 0.63-0.88 |
| **5** | **-21694.726** | 0.66 | 43517.451 | 43960.867 | 43757.489 | 56.643 (0.75) | **1** | **0.64-0.84** |
| 6 | -21671.153 | 0.69 | **43496.306** | 44029.790 | 43785.101 | 46.743 (0.25) | 1 | 0.63-0.85 |

AIC, Akaike Information Criterion; BIC, Bayesian Information Criterion; SSABIC, Sample-size adjusted Bayesian Information Criterion; LMR-LRT, Lo-Mendell-Rubin adjusted Likelihood Ratio Test; BVR, bivariate residuals. N/A, not applicable. Bold values indicate the best criterion for each model fit indicator.

**Supplemental Table 4.** Multinomial logistic regression showing the associations between mental health problem/hazardous drinking status and the latent classes of multimorbidity (N=7107). Reference group: No MHP & No HD.

|  | ***‘Physically Healthy’ class***  ***(n= 5078)*** | ***‘Emerging Multimorbidity’ class***  ***(n= 256)*** | | ***‘Hypertension & Arthritis’ class***  ***(n=1266)*** | | ***‘Digestive and Bowel Problems’ class***  ***(n= 257)*** | | ***‘Complex Multimorbidity’ class***  ***(n=250)*** | | |
| --- | --- | --- | --- | --- | --- | --- | --- | --- | --- | --- |
| **MHP/HD status** | **N (%)** | **N (%)**  **MOR (95% CI)** | **AMOR (95% CI)** | **N (%)**  **MOR (95% CI)** | **AMOR (95% CI)** | **N (%)**  **MOR (95% CI)** | **AMOR (95% CI)** | **N (%)**  **MOR (95% CI)** | **AMOR (95% CI)** |  |
| **NO MHP & NO HD**  **(n=4517)** | 3282  (72.66) | 135 (2.99)  1.00 | 1.00 | 812 (17.98)  1.00 | 1.00 | 146 (3.23)  1.00 | 1.00 | 142 (3.14)  1.00 | 1.00 |  |
| **MHP & NO HD**  **(n=1338)** | 893  (66.74) | 72 (5.38) **1.96*****  **(1.46-2.63)** | **2.24*****  **(1.65-3.03)** | 246 (18.39)  1.11  (0.95-1.31) | **1.34*****  **(1.13-1.59)** | 58 (4.33)  **1.46***  **(1.07-2.00)** | **1.58*****  **(1.15-2.17)** | 69 (5.16)  **1.79*****  **(1.33-2.40)** | **2.02*****  **(1.49-2.74)** |  |
| **NO MHP & HD**  **(n=817)** | 616  (75.4) | 27 (3.3)  1.07  (0.70-1.63) | 1.17  (0.76-1.81) | 129 (15.79)  0.85  (0.69-1.04) | 1.06  (0.85-1.31) | 27 (3.3)  0.99  (0.65-1.50) | 1.12  (0.73-1.74) | 18 (2.2)  0.68  (0.41-1.11) | 0.98  (0.59-1.62) |  |
| **MHP & HD**  **(n=435)** | 287  (65.98) | 22 (5.06) **1.86****  **(1.17-2.97)** | **2.25*****  **(1.39-3.65)** | 79 (18.16)  1.11  (0.86-1.44) | **1.63*****  **(1.23-2.16)** | 26 (5.98)  **2.04*****  **(1.32-3.14)** | **2.64*****  **(1.68-4.15)** | 21 (4.83)  **1.69***  **(1.05-2.72)** | **2.62*****  **(1.61-4.23)** |  |

MHP: Mental Health Problem; HD: Hazardous Drinking (AUDIT Score ≥8); *p<.05; **p≤.01; ***p≤.005; MOR: Multinomial Odds Ratio; AMOR: Adjusted MOR; Percentages are weighted with APMS survey weights. There were 328 missing cases in the AUDIT data, 108 missing cases in the mental health data, and three missing cases in the NCD data.

**Supplemental Table 5.** Multinomial logistic regression showing the associations between CMD/SMI and the latent classes of multimorbidity (CMD N=7543; SMI N=7091 – SMI).

|  | ***‘Physically Healthy’ class***  ***(n=5364)*** | ***‘Emerging Multimorbidity’ class***  ***(n=273)*** | | ***‘Hypertension & Arthritis’ class***  ***(n=1358)*** | | ***‘Digestive and Bowel Problems’ class***  ***(n=274)*** | | ***‘Complex Multimorbidity’ class***  ***(n=274)*** | | |
| --- | --- | --- | --- | --- | --- | --- | --- | --- | --- | --- |
|  | **N (%)** | **N (%)**  **MOR (95% CI)** | **AMOR (95% CI)** | **N (%)**  **MOR (95% CI)** | **AMOR (95% CI)** | **N (%)**  **MOR (95% CI)** | **AMOR (95% CI)** | **N (%)**  **MOR (95% CI)** | **AMOR (95% CI)** |  |
| **No CMD**  **(n=6165)** | 4494 (72.91) | 187 (3.03)  1.00 | 1.00 | 1086 (17.62)  1.00 | 1.00 | 204 (3.31)  1.00 | 1.00 | 193 (3.13)  1.00 | 1.00 |  |
| **CMD**  **(n=1381)** | 870 (63.09) | 86 (6.08)  **2.38*** (1.82-3.10)** | **2.68*** (2.03-3.53)** | 272 (19.56)  **1.29*** (1.11-1.51)** | **1.54*** (1.30-1.81)** | 70 (5.08)  **1.77*** (1.34-2.35)** | **1.85*** (1.39-2.46)** | 81 (5.87)  **2.17*****  **(1.66-2.84)** | **2.41*** (1.82-3.19)** |  |
|  | ***(n=5070)*** | ***(n=256)*** | | ***(n=1259)*** | | ***(n=260)*** | | ***(n=246)*** |  |  |
| **No SMI**  **(n=6893)** | 4947 (71.80) | 244 (3.54)  1.00 | 1.00 | 1218 (17.68)  1.00 | 1.00 | 246 (3.57)  1.00 | 1.00 | 235 (3.41)  1.00 | 1.00 |  |
| **SMI**  **(n=201)** | 123 (61.19) | 12 (5.97)  **1.98* (1.08-3.63)** | **2.22* (1.20-4.12)** | 41 (20.40)  1.35 (0.95-1.94) | **1.55* (1.05-2.27)** | 14 (6.97)  **2.29*** (1.30-4.04)** | **2.53*** (1.42-4.53)** | 11 (5.47)  **1.88***  **(1.00-3.545)** | **2.06* (1.08-3.95)** |  |

CMD: Common Mental Disorder (depression, anxiety, phobia, PTSD); SMI: Severe Mental Illness (bipolar disorder, psychotic disorder); *p<.05; **p≤.01; ***p≤.005; MOR: Multinomial Odds Ratio; AMOR: Adjusted MOR; Reference category: No CMD and no SMI. Baseline category: ‘Physically healthy’ class. Percentages are weighted with APMS survey weights. There were three missing cases in physical health conditions and 452 missing cases in the SMI data.

**Supplemental Table 6.** STROBE Checklist.

STROBE Statement—Checklist of items to be included in cross-sectional study reports.

|  | **Item No** | **Recommendation** | **Page No** |
| --- | --- | --- | --- |
| **Title and abstract** | 1 | (*a*) Indicate the study’s design with a commonly used term in the title or the abstract | 1  (Abstract) |
|  |  | (*b*) Provide in the abstract an informative and balanced summary of what was done and what was found | 1 |
| **Introduction** | | | |
| Background/rationale | 2 | Explain the scientific background and rationale for the investigation being reported | 3, 4 |
| Objectives | 3 | State specific objectives, including any prespecified hypotheses | 4, 5 |
| **Methods** | | | |
| Study design | 4 | Present key elements of study design early in the paper | 5 |
| Setting | 5 | Describe the setting, locations, and relevant dates, including periods of recruitment, exposure, follow-up, and data collection | 5 (reference to previous article) |
| Participants | 6 | (*a*) Give the eligibility criteria, and the sources and methods of selection of participants | 5 (reference to previous article) |
| Variables | 7 | Clearly define all outcomes, exposures, predictors, potential confounders, and effect modifiers. Give diagnostic criteria, if applicable | 6, 7, 8 |
| Data sources/ measurement | 8* | For each variable of interest, give sources of data and details of methods of assessment (measurement). Describe comparability of assessment methods if there is more than one group | 6, 7, 8 |
| Bias | 9 | Describe any efforts to address potential sources of bias | 8 |
| Study size | 10 | Explain how the study size was arrived at | 5 (reference to previous article) |
| Quantitative variables | 11 | Explain how quantitative variables were handled in the analyses. If applicable, describe which groupings were chosen and why | 6, 7 |
| Statistical methods | 12 | (*a*) Describe all statistical methods, including those used to control for confounding | 8, 9 |
|  |  | (*b*) Describe any methods used to examine subgroups and interactions | 8, 9 |
|  |  | (*c*) Explain how missing data were addressed | 8 |
|  |  | (*d*) If applicable, describe analytical methods taking account of sampling strategy | 8 |
|  |  | (*e*) Describe any sensitivity analyses | 9 |
| **Results** | | | |
| Participants | 13* | (a) Report numbers of individuals at each stage of study—eg numbers potentially eligible, examined for eligibility, confirmed eligible, included in the study, completing follow-up, and analysed | 9, |
|  |  | (b) Give reasons for non-participation at each stage |  |
|  |  | (c) Consider use of a flow diagram |  |
| Descriptive data | 14* | (a) Give characteristics of study participants (eg demographic, clinical, social) and information on exposures and potential confounders | 9, 10, 12 |
|  |  | (b) Indicate number of participants with missing data for each variable of interest | 9, 10 |
| Outcome data | 15* | Report numbers of outcome events or summary measures |  |
| Main results | 16 | (*a*) Give unadjusted estimates and, if applicable, confounder-adjusted estimates and their precision (eg, 95% confidence interval). Make clear which confounders were adjusted for and why they were included | 13, 14 |
|  |  | (*b*) Report category boundaries when continuous variables were categorized |  |
|  |  | (*c*) If relevant, consider translating estimates of relative risk into absolute risk for a meaningful time period |  |
| Other analyses | 17 | Report other analyses done—eg analyses of subgroups and interactions, and sensitivity analyses | 14 |
| **Discussion** | | | |
| Key results | 18 | Summarise key results with reference to study objectives | 15 |
| Limitations | 19 | Discuss limitations of the study, taking into account sources of potential bias or imprecision. Discuss both direction and magnitude of any potential bias | 17, 18 |
| Interpretation | 20 | Give a cautious overall interpretation of results considering objectives, limitations, multiplicity of analyses, results from similar studies, and other relevant evidence | 17, 18 |
| Generalisability | 21 | Discuss the generalisability (external validity) of the study results | 18 |
| **Other information** | | | |
| Funding | 22 | Give the source of funding and the role of the funders for the present study and, if applicable, for the original study on which the present article is based | 19 |

*Give information separately for exposed and unexposed groups.

**Note:** An Explanation and Elaboration article discusses each checklist item and gives methodological background and published examples of transparent reporting. The STROBE checklist is best used in conjunction with this article (freely available on the Web sites of PLoS Medicine at http://www.plosmedicine.org/, Annals of Internal Medicine at http://www.annals.org/, and Epidemiology at http://www.epidem.com/). Information on the STROBE Initiative is available at www.strobe-statement.org.
